# Supplementary material for: Fluorescence-Responsive Detection of Ag(I), Al(III), and Cr(III) Ions Using Cd(II) Based Pillared-Layer Frameworks
Source: Int J Mol Sci. 2022 Dec 26;24(1):369. doi: 10.3390/ijms24010369 (PMC9820227; doi:10.3390/ijms24010369)
Supplement: Supplementary file 1 [file ijms-24-00369-s001.zip › ijms-2081548-supplementary.pdf]

Supporting Information

Fluorescence-Responsive Detection of Ag(I), Al(III), and Cr(III) Ions Using Cd(II)-Based Pillared-Layer Frameworks

Qi-Jin Jiang, Po-Min Chuang, and Jing-Yun Wu*

Department of Applied Chemistry, National Chi Nan University, Nantou 545, Taiwan.

* Correspondence: jyunwu@ncnu.edu.tw (J.-Y.W.)

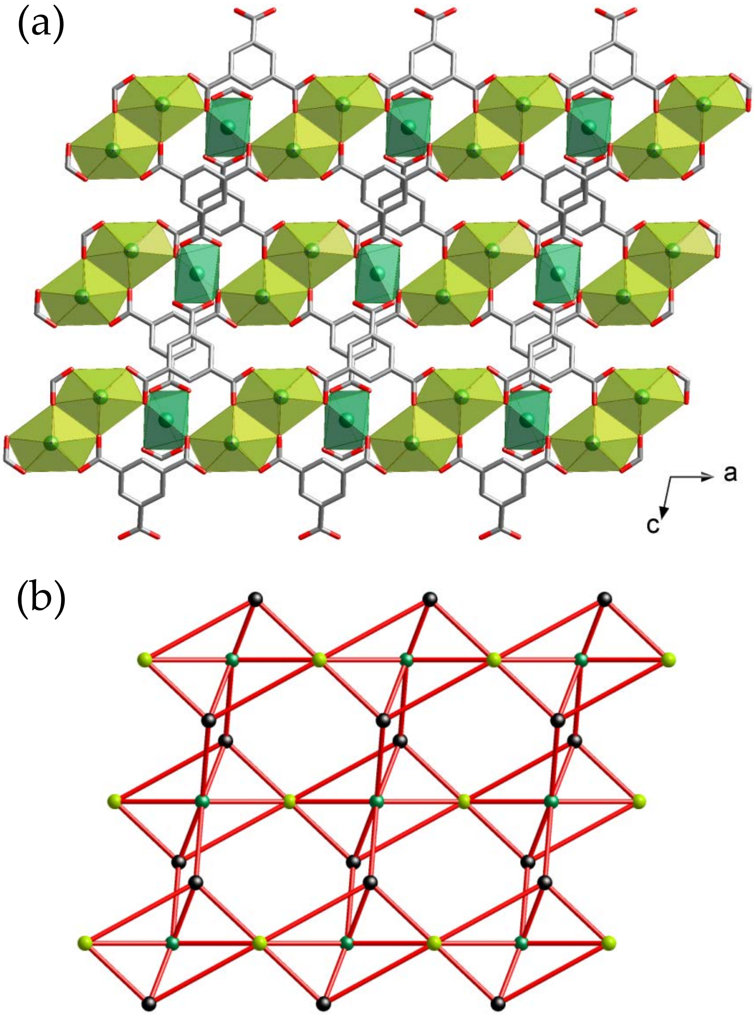

Figure S1. (a) Polyhedral and (b) schematic representations of the two-dimensional $[Cd_2(btc)_3]_n$ bilayer structure of **1** showing the (4,6,6)-connected net topology.

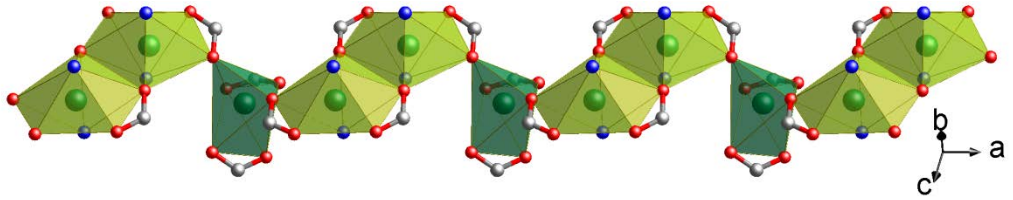

Figure S2. The one-dimensional $\{Cd_3(CO_2)_6\}_n$ chains running along the [100] direction in **1**.

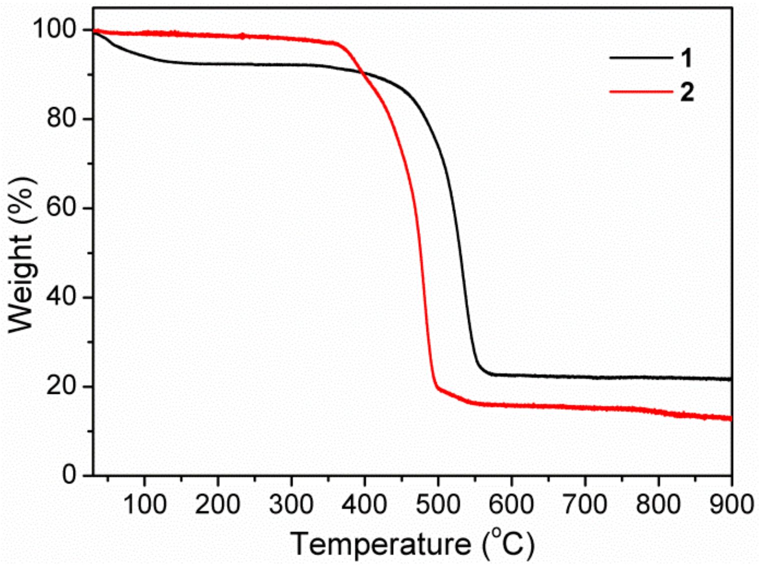

Figure S3. TG curves of **1** and **2**.

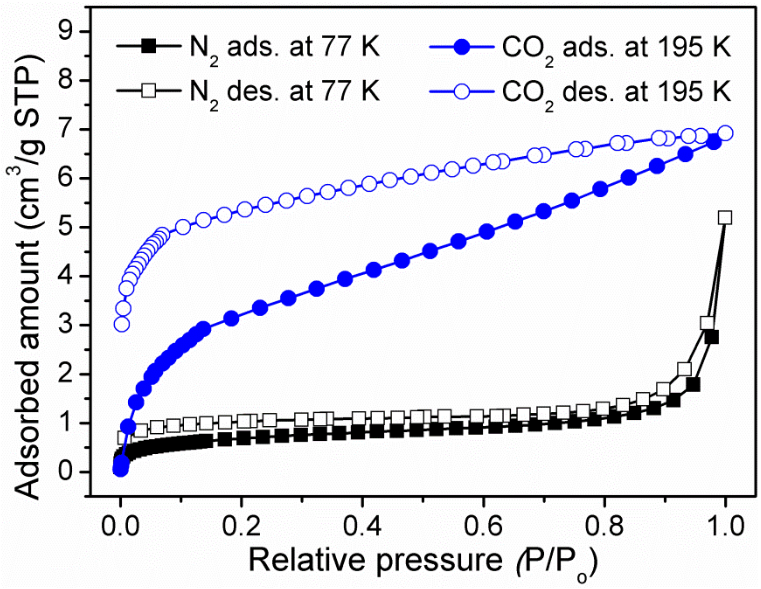

Figure S4. N_2 and CO_2 adsorption isotherms of thermally activated **1**.

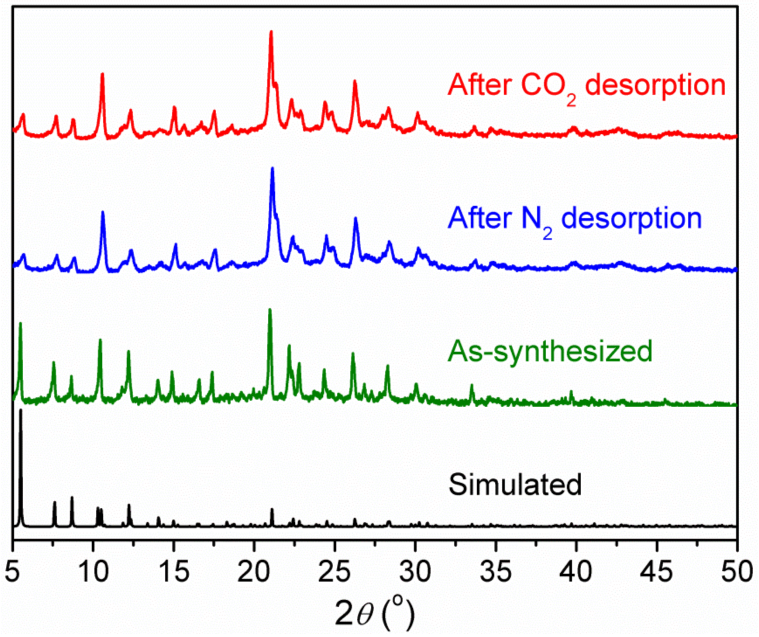

Figure S5. XRPD patterns of **1** before and after N_2 and CO_2 adsorption−desorption.

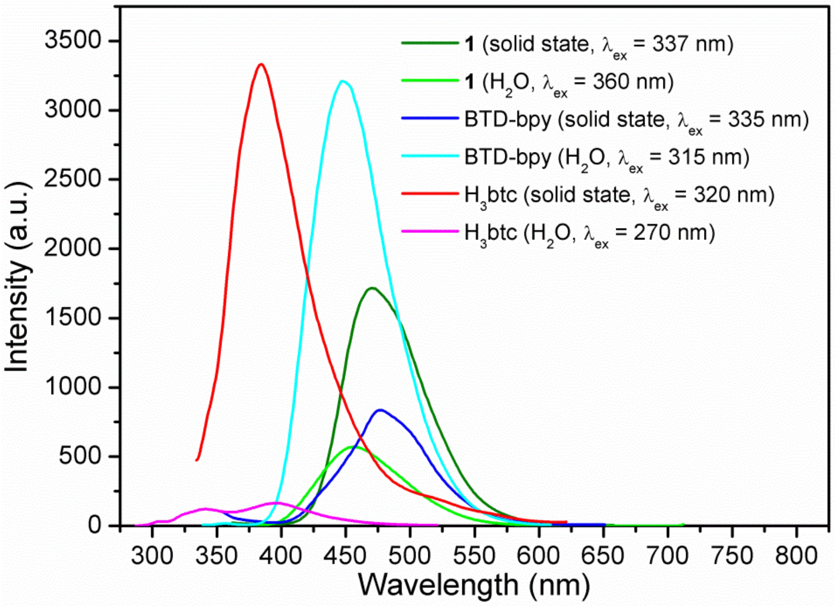

Figure S6. Emission spectra of **1**, BTD-bpy and H_3btc in solid-state and in H_2O at room temperature.

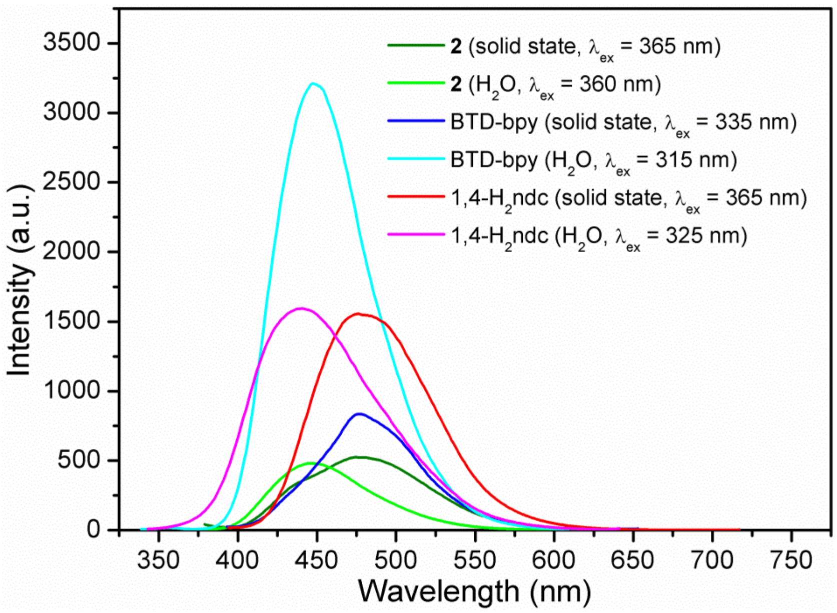

Figure S7. Emission spectra of **2**, BTD-bpy and 1,4-H_2ndc in solid-state and in H_2O at room temperature.

(a)

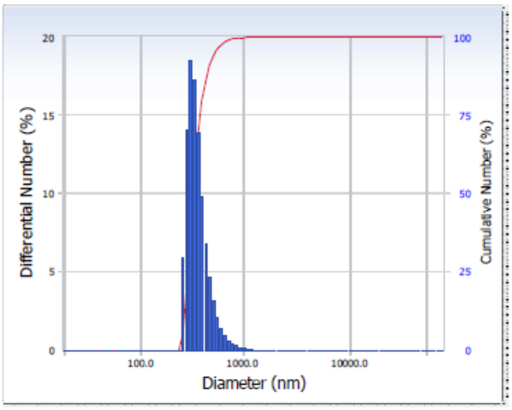

Distribution Results (Contin)

Peak	Diameter (nm)	Std. Dev.
1	372.3	102.9
2	0.0	0.0
3	0.0	0.0
4	0.0	0.0
5	0.0	0.0
Average	372.3	102.9

(b)

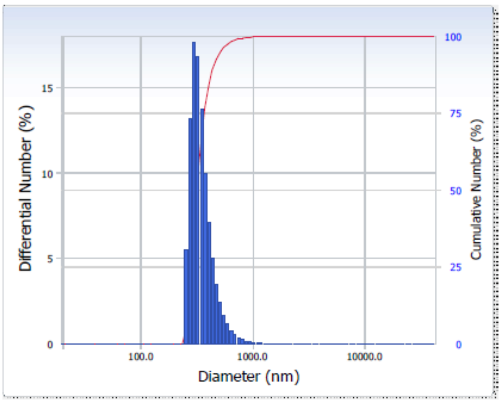

Distribution Results (Contin)

Peak	Diameter (nm)	Std. Dev.
1	362.2	100.2
2	0.0	0.0
3	0.0	0.0
4	0.0	0.0
5	0.0	0.0
Average	362.2	100.2

Figure S8. Particle size distributions of (a) **1** and (b) **2**.

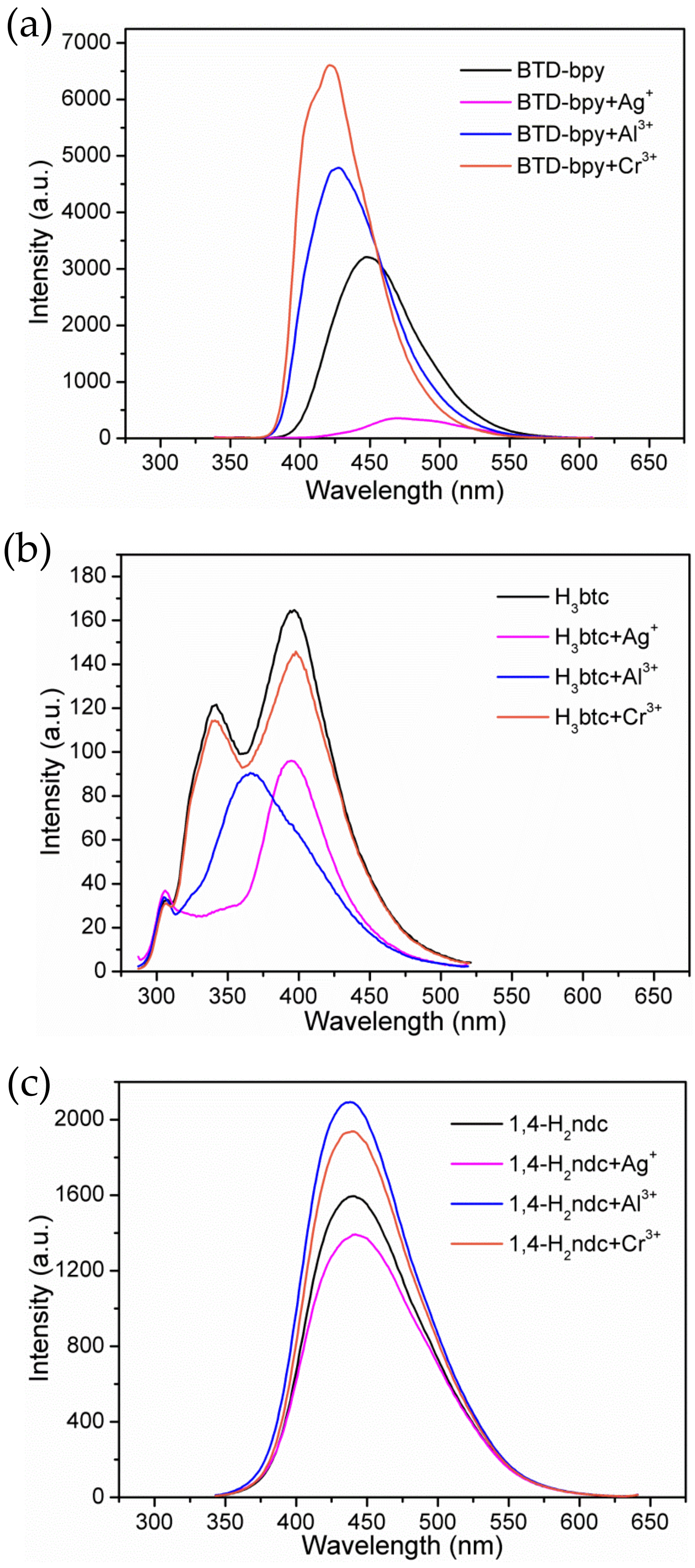

Figure S9. Emission spectra of (a) BTD-bpy, (b) H_3btc, and (c) 1,4-H_2ndc in H_2O (1 mg/3 mL) before and after addition of Ag^{+}, Al^{3+}, and Cr^{3+} ions (1.0 mM).

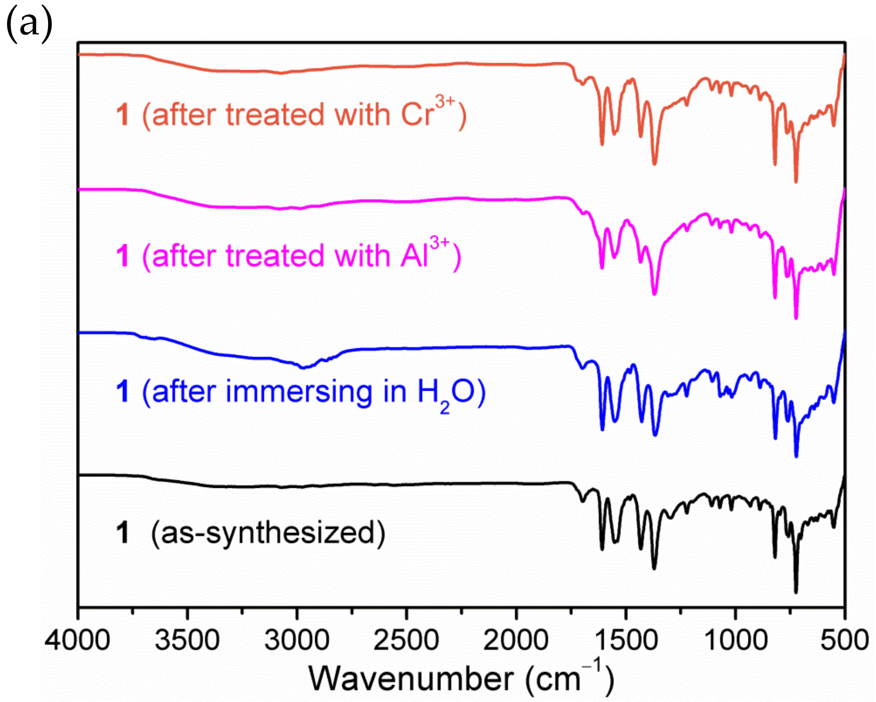

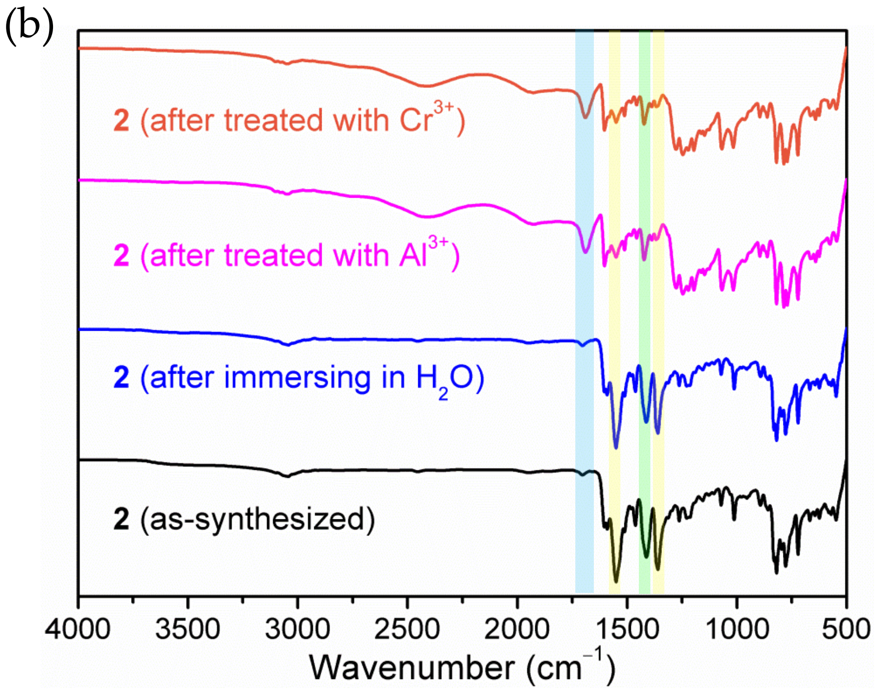

Figure S10. (a) IR spectra of **1**: as-synthesized, after immersion in H_2O for 1 day, after treated with Al^{3+} and Cr^{3+} in H_2O for 1 day. (b) IR spectra of **2**: as-synthesized, after immersion in H_2O for 1 day, after treated with Al^{3+} and Cr^{3+} in H_2O for 1 day.

Table S1. Comparison of literature reports for Ag^{+} detection using CP/MOF-based luminescence chemosensors.

Luminescence chemosensors	Analyte	Solvent	Mechanism	DL, μM	Ref.
$Sm_8(HDBA)_6{\cdot}H_2O$ (Sm-MOF)	Ag^+	H_2O	Turn-on	3.95	S1
Eu^{3+}@Ga(OH)(btec)·0.5H_2O (Eu-MIL-61)	Ag^+	H_2O	Turn-on	0.23	S2
Sm^{3+}@(Ga(OH)(btec)·0.5H_2O (Sm-MIL-61)	Ag^+	H_2O	Turn-on	0.03	S3
Eu3$^+$@Al(OH)(H_2btec)·H_2O (Eu^{3+}@MIL-121)	Ag^+	H_2O	Turn-on	0.1	S4
Sm^{3+}@Al(OH)(H_2btec)·H_2O (Sm^{3+}@MIL-121)	Ag^+	H_2O	Turn-on	0.09	S5
Eu^{3+}@$C_{12}H_6N_2O_{5.33}Zr$ (Eu^{3+}@UiO-67)	Ag^+	H_2O	Turn-off	0.049	S6
$[Co(DCTP)(L^1)(H_2O)_2]_n$ (NCST-1)	Ag^+	H_2O	Turn-off	1.33	S7
$\{[Mn_2(Hbtc)_2(L^2)_2(H_2O)_2]{\cdot}2H_2O\}_n$ (NCST-4)	Ag^+	EtOH	Turn-off	0.67	S8
$\{Cd_3(btc)_2$(BTD-bpy)$_2]{\cdot}1.5MeOH{\cdot}4H_2O\}_n$ (**1**)	Ag^+	H_2O	Turn-off	0.56	This work
$[Cd_2$(1,4-ndc)$_2$(BTD-bpy)$_2]_n$ (**2**)	Ag^+	H_2O	Turn-off	1.47	This work

Abbreviations: H_5DBA = 3,5-di(2′,4′-dicarboxylphenyl)benzoic acid; H_4btec = pyromellitic acid; H_2DCTP = 2,5-dichloroterephthalic aci;d L^1 = 1,4-bis(benzimidazol-1-ylmethyl)benzene; H_3btc = benzene-1,3,5-tricarboxylic acid; L^2 = 1,4-bis(5,6-dimethylbenzimidazole)butane; BTD-bpy = bis(pyridin-4-yl)benzothiadiazole; 1,4-H_2ndc = naphthalene-1,4-dicarboxylic acid.

Table S2. Comparison of literature reports for Al^{3+} detection using CP/MOF-based fluorescence chemosensors in water.

Fluorescence chemosensors	Analyte	Mechanism	DL, μM	Ref.
$\{[Ba_3La_{0.5}(L^3)_{2.5}(H_2O)_3(DMF)]\}_n$	Al^{3+}	Turn-off	1.11	S9
$[Cd(PAM)(4\text{-bpdb})_{1.5}]\cdot DMF$	Al^{3+}	Turn-off	0.56	S10
$[Zn(5\text{-}NH_2\text{-}1,3\text{-bdc})(H_2O)]$	Al^{3+}	Turn-off	2.44	S11
$[Zn_2(5\text{-}NH_2\text{-}1,3\text{-bdc})_2(NI\text{-bpy-}44)]\cdot DMF$	Al^{3+}	Turn-off	5.59	S11
$[Eu_2(ppda)_2(npdc)(H_2O)]\cdot H_2O$	Al^{3+}	Turn-off	109	S12
$[Zn_2(OH)(1,4\text{-ndc})_{1.5}(Cz\text{-}3,6\text{-bpy})]\cdot 2H_2O$	Al^{3+}	Ratiometric	9.97	S13
$\{[Zn_2(OH)(Br\text{-}1,4\text{-bdc})_{1.5}(Cz\text{-}3,6\text{-bpy})]\cdot H_2O\}_n$	Al^{3+}	Ratiometric turn-off	0.59	S14
$\{[Zn_2(OH)(Br\text{-}1,4\text{-bdc})_{1.5}(Cz\text{-Pr-}3,6\text{-bpy})]\cdot 0.5H_2O\}_n$	Al^{3+}	Ratiometric turn-off	1.89	S14
$\{[Zn_2(OH)(NO_2\text{-}1,4\text{-bdc})_{1.5}(Cz\text{-}3,6\text{-bpy})]\cdot 2H_2O\}_n$	Al^{3+}	Ratiometric turn-on	4.74	S14
$\{[Zn_2(OH)(NO_2\text{-}1,4\text{-bdc})_{1.5}(Cz\text{-Pr-}3,6\text{-bpy})]\cdot 0.5H_2O\}_n$	Al^{3+}	Ratiometric turn-on	5.90	S14
$[Co(OBA)(DATZ)_{0.5}(H_2O)]$	Al^{3+}	Turn-on	2.5	S15
$[Co(H_2L)(TPY)(H_2O)]\cdot H_2O$	Al^{3+}	Turn-on	1.67	S16
Zn(DMA)(TBA)	Al^{3+}	Turn-on	1.97	S17
$[Zn(H_2dhbdc)(Cz\text{-}3,6\text{-bpy})]_n$	Al^{3+}	Turn-on	0.62	S18
$[Cd(H_2dhbdc)(NI\text{-mbpy-}34)_2]_n$	Al^{3+}	Turn-on	0.34	S19
$\{[Cd(Br\text{-}1,3\text{-bdc})(NI\text{-mbpy-}34)(H_2O)]\cdot 2H_2O\}_n$	Al^{3+}	Turn-on	3.37	S20
$\{Cd_3(btc)_2(BTD\text{-bpy})_2]\cdot 1.5MeOH\cdot 4H_2O\}_n$ (**1**)	Al^{3+}	Turn-on	4.97	This work
$[Cd_2(1,4\text{-ndc})_2(BTD\text{-bpy})_2]_n$ (**2**)	Al^{3+}	Turn-on	0.25	This work

Abbreviations: H_3L^3 = p-terphenyl-3,4″,5-tricarboxylic acid; DMF = *N,N*'-dimethylformamide; PAM = 4,4'-methylenebis(3-hydroxy-2-naphthalene-carboxylic acid; 4-bpdb = 1,4-bis(4-pyridyl)-2,3-diaza-1,3-butadiene; 5-NH_2-1,3-H_2bdc = 5-amino-1,3-benzenedicarboxylic acid; NI-bpy-44 = *N*-(pyridin-4-yl)-4-(pyridin-4-yl)-1,8-naphthalimide; H_2ppda = 4-(pyridine-3-yloxy)phthalic acid; H_2npdc = naphthalene-1,4-dicarboxylic acid; 1,4-H_2ndc = naphthalene-1,4-dicarboxylic acid; Cz-3,6-bpy = 3,6-bis(pyridin-4-yl)-9*H*-carbazole; Br-1,4-H_2bdc = 2-bromobenzene-1,4-dicarboxylic acid; Cz-Pr-3,6-bpy = 3,6-bis(pyridin-4-yl)-9-*n*-propyl-9*H*-carbazole; NO_2-1,4-H_2bdc = 2-nitrobenzene-1,4-dicarboxylic acid; DMA = *N,N*'-dimethylacetamide; H_2TBA = 4-(1H-tetrazol-5-yl)-benzoic acid; H_2OBA = 4,4'-oxybis(benzoic acid); DATZ = 3,5-diamino-1,2,4-triazole; H_4L = [1,1′:4′,1″-terphenyl]-2′,4,4″,5′-tetracarboxylic acid; TPY = 4,3′:5′,4″-terpyridine; H_4dhbdc = 2,5-dihydroxyterephthalic acid; NI-mbpy-34 = *N*-(pyridin-3-ylmethyl)-4-(pyridin-4-yl)-1,8-naphthalimide; Br-1,3-H_2bdc = 5-bromobenzene-1,3-dicarboxylic acid; H_3btc = benzene-1,3,5-tricarboxylic acid; BTD-bpy = bis(pyridin-4-yl)benzothiadiazole.

Table S3. Comparison of literature reports for Cr^{3+} detection using CP/MOF-based fluorescence chemosensors in water.

Fluorescence Chemosensors	Analyte	Mechanism	DL, μM	Ref.
$[Zn(5\text{-}NH_2\text{-}1,3\text{-}bdc)(H_2O)]$	Cr^{3+}	Turn-off	13.9	S11
$[Zn_2(5\text{-}NH_2\text{-}1,3\text{-}bdc)_2(NI\text{-}bpy\text{-}44)]\cdot DMF$	Cr^{3+}	Turn-off	7.87	S11
$[Zn(L^4)(H_2O)]\cdot H_2O$	Cr^{3+}	Turn-off	2.44	S21
$[Eu_2(ppda)_2(npdc)(H_2O)]\cdot H_2O$	Cr^{3+}	Turn-off	61.7	S12
$\{[Eu_3(bpydb)_3(HCOO)(OH)_2(DMF)]\cdot 3DMF\cdot 2H_2O\}_n$	Cr^{3+}	Turn-off	1.0	S22
$[Zn_2(OH)(1,4\text{-}ndc)_{1.5}(Cz\text{-}3,6\text{-}bpy)]\cdot 2H_2O$	Cr^{3+}	Ratiometric	8.61	S13
$\{[Zn_2(OH)(Br\text{-}1,4\text{-}bdc)_{1.5}(Cz\text{-}3,6\text{-}bpy)]\cdot H_2O\}_n$	Cr^{3+}	Ratiometric turn-off	0.49	S14
$\{[Zn_2(OH)(Br\text{-}1,4\text{-}bdc)_{1.5}(Cz\text{-}Pr\text{-}3,6\text{-}bpy)]\cdot 0.5H_2O\}_n$	Cr^{3+}	Ratiometric turn-off	2.18	S14
$\{[Zn_2(OH)(NO_2\text{-}1,4\text{-}bdc)_{1.5}(Cz\text{-}3,6\text{-}bpy)]\cdot 2H_2O\}_n$	Cr^{3+}	Ratiometric turn-on	3.03	S14
$\{[Zn_2(OH)(NO_2\text{-}1,4\text{-}bdc)_{1.5}(Cz\text{-}Pr\text{-}3,6\text{-}bpy)]\cdot 0.5H_2O\}_n$	Cr^{3+}	Ratiometric turn-on	3.22	S14
$[Cd(H_2dhbdc)(NI\text{-}mbpy\text{-}34)_2]_n$	Cr^{3+}	Turn-on	1.47	S19
$\{[Cd(Br\text{-}1,3\text{-}bdc)(NI\text{-}mbpy\text{-}34)(H_2O)]\cdot 2H_2O\}_n$	Cr^{3+}	Turn-on	2.81	S20
$[Zn(Br\text{-}1,3\text{-}bdc)(NI\text{-}mbpy\text{-}34)]_n$	Cr^{3+}	Turn-on	3.13	S23
$\{[Cd_2(adc)_2(4\text{-}nvp)_6]\cdot(MeOH)\cdot(H_2O)\}_n$	Cr^{3+}	Turn-on	0.31	S24
$\{Cd_3(btc)_2(BTD\text{-}bpy)_2]\cdot 1.5MeOH\cdot 4H_2O\}_n$ (**1**)	Cr^{3+}	Turn-on	3.03	This work
$[Cd_2(1,4\text{-}ndc)_2(BTD\text{-}bpy)_2]_n$ (**2**)	Cr^{3+}	Turn-on	0.79	This work

Abbreviations: $5\text{-}NH_2\text{-}1,3\text{-}H_2bdc$ = 5-amino-1,3-benzenedicarboxylic acid; NI-bpy-44 = *N*-(pyridin-4-yl)-4-(pyridin-4-yl)-1,8-naphthalimide; DMF = *N*,*N*'-dimethylformamide; H_2L^4 = 5-(2-methylpyridin-4-yl)isophthalic acid; H_2ppda = 4-(pyridine-3-yloxy)phthalic acid; H_2npdc = naphthalene-1,4-dicarboxylic acid; $bpydbH_2$ = 4,4'-(4,4' -bipyridine-2,6-diyl) dibenzoic acid; $1,4\text{-}H_2ndc$ = naphthalene-1,4-dicarboxylic acid; Cz-3,6-bpy = 3,6-bis(pyridin-4-yl)-9*H*-carbazole; $Br\text{-}1,4\text{-}H_2bdc$ = 2-bromobenzene-1,4-dicarboxylic acid; Cz-Pr-3,6-bpy = 3,6-bis(pyridin-4-yl)-9-*n*-propyl-9*H*-carbazole; $NO_2\text{-}1,4\text{-}H_2bdc$ = 2-nitrobenzene-1,4-dicarboxylic acid; H_4dhbdc = 2,5-dihydroxyterephthalic acid; NI-mbpy-34 = *N*-(pyridin-3-ylmethyl)-4-(pyridin-4-yl)-1,8-naphthalimide; $Br\text{-}1,3\text{-}H_2bdc$ = 5-bromobenzene-1,3-dicarboxylic acid; H_2adc = 9,10-anthracenedicarboxylic acid; 4-nvp = 4-(1-naphthylvinyl)pyridine; H_3btc = benzene-1,3,5-tricarboxylic acid; BTD-bpy = bis(pyridin-4-yl)benzothiadiazole.

References

(S1) Zhang, G.-q.; Gao, L.-j.; Chai, H.-m.; Ren, Y.-x. Novel Multifunctional Samarium−Organic Framework for Fluorescence Sensing of Ag^+, MnO_4^-, and Cimetidine and Electrochemical Sensing of o-Nitrophenol in Aqueous Solutions. *ACS Omega* **2021**, *6*, 6810−6816.

(S2) Sun, N.; Yan, B. A reliable amplified fluorescence-enhanced chemosensor (Eu-MIL-61) for the directional detection of Ag^+ in an aqueous solution. *Dalton Trans.* **2017**, *46*, 875−881.

(S3) Sun, N.; Yan, B. Ag^+-induced photoluminescence enhancement in lanthanide post-functionalized MOFs and Ag^+ sensing. *Phys. Chem. Chem. Phys.* **2017**, *19*, 9174−9180.

(S4) Hao, J.-N.; Yan, B. Highly sensitive and selective fluorescent probe for Ag^+ based on a Eu^{3+} post-functionalized metal−organic framework in aqueous media. *J. Mater. Chem. A* **2014**, *2*, 18018−18025.

(S5) Hao, J.-N.; Yan, B. Ag^+-sensitized lanthanide luminescence in Ln^{3+} post-functionalized metal−organic frameworks and Ag^+ sensing. *J. Mater. Chem. A* **2015**, *3*, 4788−4792.

(S6) Luo, J.; Liu, B.-S.; Zhang, X.-R.; Liu, R.-T. A new fluorescent sensor constructed by Eu^{3+} post-functionalized metal−organic framework for sensing Ag^+ with high selectivity and sensitivity in aqueous solution. *J. Mol. Struct.* **2021**, *1227*, 129518.

(S7) Xiao, Q.-Q.; Liu, D.; Wei, Y.-L.; Cui, G.-H. A new multifunctional two-dimensional cobalt(II) metal−organic framework for electrochemical detection of hydrogen peroxide, luminescent sensing of metal ions, and photocatalysis. *Polyhedron* **2019**, *158*, 342−351.

(S8) Xiao, Q.-Q.; Liu, D.; Wei, Y.-L.; Cui, G.-H. Two new ternary Mn(II) coordination polymers by regulation of aromatic carboxylate ligands: Synthesis, structures, photocatalytic and selective ion-sensing properties. *J. Solid State Chem.* **2019**, *273*, 67−74.

(S9) Ding, B.; Liu, S. X.; Cheng, Y.; Guo, C.; Wu, X. X.; Guo, J. H.; Liu, Y. Y.; Li, Y. Heterometallic Alkaline Earth−Lanthanide Ba^{II}−La^{III} Microporous Metal−Organic Framework as Bifunctional Luminescent Probes of Al^{3+} and MnO_4^-. *Inorg. Chem.* **2016**, *55*, 4391−4402.

(S10) Lv, R.; Chen, Z.; Fu, X.; Yang, B.; Li, H.; Su, J.; Gu, W.; Liu, X. A Highly Selective and Fast-Response Fluorescent Probe Based on Cd-MOF for the Visual Detection of Al^{3+} Ion and Quantitative Detection of Fe^{3+} Ion. *J. Solid State Chem.* **2018**, *259*, 67–72.

(S11) Zhang, J.-R.; Lee, J.-J.; Su, C.-H.; Tsai, M.-J.; Li, C.-Y.; Wu, J.-Y. From lamellar net to bilayered-lamella and to porous pillared-bilayer: reversible crystal-to-crystal transformation, CO_2 adsorption, and fluorescence detection of Fe^{3+}, Al^{3+}, Cr^{3+}, MnO_4^-, and $Cr_2O_7^{2-}$ in water. *Dalton Trans.* **2020**, *49*, 14201–14215.

(S12) Zhan, Z.; Liang, X.; Zhang, X.; Jia, Y.; Hu, M. A water-stable europium-MOF as a multifunctional luminescent sensor for some trivalent metal ions (Fe^{3+}, $Cr3^+$, Al^{3+}), PO_4^{3-} ions, and nitroaromatic explosives. *Dalton Trans.* **2019**, *48*, 1786–1794.

(S13) Chuang, P.-M.; Wu, J.-Y. A highly stable Zn coordination polymer exhibiting pH-dependent fluorescence and as a visually ratiometric and on–off fluorescence sensor. *CrystEngComm* **2021**, *23*, 5226–5240.

(S14) Chuang, P.-M.; Huang, Y.-W.; Liu, Y.-L.; Wu, J.-Y. Influence of linker substitution on fluorescence responsive sensing of isostructural coordination polymers: visual turn-on, ratiometric, and turn-off sensing in water. *CrystEngComm* **2021**, *23*, 2222–2234.

(S15) Singha, D. T.; Mahata, P. Highly Selective and Sensitive Luminescence Turn-On-Based Sensing of Al^{3+} Ions in Aqueous Medium Using a MOF with Free Functional Sites. *Inorg. Chem.* **2015**, *54*, 6373–6379.

(S16) Zhang, C.-L.; Liu, Z.-T.; Xu, H.; Zheng, H.-G.; Ma, J.; Zhao, J. An excellent example illustrating the fluorescence sensing property of cobalt–organic frameworks. *Dalton Trans.* **2019**, *48*, 2285–2289.

(S17) Zhang, X.; Luo, X.; Zhang, N.; Wu, J.; Huang, Y.-Q. A highly selective and sensitive Zn(II) coordination polymer luminescent sensor for Al^{3+} and NACs in the aqueous phase. *Inorg. Chem. Front.* **2017**, *4*, 1888–1894.

(S18) Chen, W.-T.; Tsai, M.-J.; Wu, J.-Y. A Thermally Stable Undulated Coordination Layer Showing a Sequentially Interweaving 2D $\rightarrow$ 3D Net as a Turn-On Sensor for Luminescence Detection of Al^{3+} in Water. *Cryst. Growth Des.* **2022**, *22*, 226–236.

(S19) Tsai, M.-J.; Liao, K.-S.; Hsu, L.-J.; Wu, J.-Y. A luminescent Cd(II) coordination polymer as a fluorescence-responsive sensor for enhancement sensing of Al^{3+} and Cr^{3+} ions and quenching detection of chromium(VI) oxyanions. *J. Solid State Chem.* **2021**, *304*, 122564.

(S20) Liao, K.-S.; Tsai, M.-J.; Hsu, L.-J.; Wang, C.-M.; Wu, J.-Y. A Cd(II) luminescent coordination grid as a multiresponsive fluorescence sensor for Cr(VI) oxyanions and Cr(III), Fe(III), and Al(III) in aqueous medium. *Molecules* **2021**, *26*, 7103.

(S21) Guo, X.-Y.; Zhao, F.; Liu, J.-J.; Liu, Z.-L.; Wang, Y.-Q. An ultrastable zinc(II)−organic framework as a recyclable multi-responsive luminescent sensor for Cr(III), Cr(VI) and 4-nitrophenol in the aqueous phase with high selectivity and sensitivity. *J. Mater. Chem. A* **2017**, *5*, 20035–20043.

(S22) Yang, S.-L.; Yuan, Y.-Y.; Sun, P.-P.; Lin, T.; Zhang, C.-X.; Wang, Q.-L. 3D water-stable europium metal organic frameworks as a multi-responsive luminescent sensor for high-efficiency detection of $Cr_2O_7^{2-}$, MnO_4^-, Cr^{3+} ions and SDBS in aqueous solution. *New J. Chem.* **2018**, *42*, 20137–20143.

(S23) Tsai, M.-J.; Liao, K.-S.; Wu, J.-Y. A Water-Stable 2-Fold Interpenetrating cds Net as a Bifunctional Fluorescence-Responsive Sensor for Selective Detection of Cr(III) and Cr(VI) Ions. *Nanomaterials* **2022**, *12*, 158.

(S24) Dutta, B.; Jana, R.; Bhanja, A. K.; Ray, P. P.; Sinha, C.; Mir, M. H. Supramolecular Aggregate of Cadmium(II)-Based One-Dimensional Coordination Polymer for Device Fabrication and Sensor Application. *Inorg. Chem.* **2019**, *58*, 2686–2694.
